# Anatomical Calibration through Post-Processing of Standard Motion Tests Data

**DOI:** 10.3390/s16122011

**Published:** 2016-11-28

**Authors:** Weisheng Kong, Salvatore Sessa, Massimiliano Zecca, Atsuo Takanishi

**Affiliations:** 1 Graduate School of Advanced Science and Engineering, Waseda University, Tokyo 169-8555, Japan; kong.ws@fuji.waseda.jp; 2 Graduate School of Creative Science and Engineering, Waseda University, Tokyo 169-8555, Japan; s.sessa@aoni.waseda.jp; 3 Wolfson School of Mechanical, Electrical and Manufacturing Engineering, Loughborough University, Loughborough LE11 3TU, UK; m.zecca@lboro.ac.uk; 4 National Centre for Sport and Exercise Medicine, Loughborough LE11 3TU, UK; 5 NIHR Leicester-Loughborough Diet, Lifestyle and Physical Activity Biomedical Research Unit, Loughborough LE11 3TU, UK; 6 Department of Modern Mechanical Engineering, Waseda University, Tokyo 169-8555, Japan; 7 Humanoid Robotics Institute (HRI), Waseda University, Tokyo 162-0044, Japan

**Keywords:** anatomical calibration, sensor-to-body alignment, functional calibration, inertial measurement unit, accelerometer, principal component analysis, motion test, walking, sit-to-stand, sagittal plane

## Abstract

The inertial measurement unit is popularly used as a wearable and flexible tool for human motion tracking. Sensor-to-body alignment, or anatomical calibration (AC), is fundamental to improve accuracy and reliability. Current AC methods either require extra movements or are limited to specific joints. In this research, the authors propose a novel method to achieve AC from standard motion tests (such as walking, or sit-to-stand), and compare the results with the AC obtained from specially designed movements. The proposed method uses the limited acceleration range on medial-lateral direction, and applies principal component analysis to estimate the sagittal plane, while the vertical direction is estimated from acceleration during quiet stance. The results show a good correlation between the two sets of IMUs placed on frontal/back and lateral sides of head, trunk and lower limbs. Moreover, repeatability and convergence were verified. The AC obtained from sit-to-stand and walking achieved similar results as the movements specifically designed for upper and lower body AC, respectively, except for the feet. Therefore, the experiments without AC performed can be recovered through post-processing on the walking and sit-to-stand data. Moreover, extra movements for AC can be avoided during the experiment and instead achieved through the proposed method.

## 1. Introduction

Inertial Measurement Units (IMUs) [1] have recently been gaining popularity in motion capture [2], feature extraction [3,4,5] and motion evaluation [6,7]. IMU systems are usually regarded as a wearable and flexible alternative [8,9] for marker-based optical tracking systems, especially in daily monitoring and outdoor measurement, because they are wearable and their workspace is not limited to a special room equipped with cameras. However, one fundamental difference between the two systems is usually not mentioned. For optical tracking systems, all the measurements are with respect to only one reference frame. Conversely, for IMU system the measurement is with respect to the coordinates frame of each sensor, and as such all different. Therefore, for analysis with data from IMUs, special attention must be paid to the alignment between the coordinates frames of different IMUs, and between IMUs and body segments. In most of the researches [3,4,5,7] this alignment was skipped or performed by manually adjusting the placement of IMUs, assuming that the axes of sensor could represent the axes of the body segment where the sensor is attached.

However, in practice it is very difficult to perfectly align the axis of the sensor with the axis of the body segment, for the following main reasons:

- First, the sensor is attached to the curved body surface so it could be difficult to find a place where the two coordinates frames are aligned.
- Second, the placement of the sensor has to avoid the main active skeletal muscle groups which further limits the possible placement area.
- Finally, the well-known sensor placement may not be feasible in some particular tests. For example, the sensor on waist is usually placed on the center of back close to L5. However, in Stand-to-Sit Test, the sensor on the back may cause pain for the subject or altered movement to avoid hitting the sensor on the back of chair.

Therefore, the alignment of coordinates frames of IMUs is needed for better measurement accuracy and reliability.

The alignment between sensor and body segment has been named in different ways across the state of the arts: sensor to segment calibration [10]; anatomical calibration [11,12]; system calibration [2]; and also functional calibration [13]. In this paper we use the term anatomical calibration. The anatomical calibration could be classified into the following categories: (1) Direct anatomical landmark identification (ALI); (2) Imposition of joint constraints (JCI); (3) Multibody modelling (MBM); (4) Functional approach (FUA).

ALI performed anatomical calibration by using extra devices such as an IMU embedded caliper [12] or a camera-mounted L-shaped stick [14]. The device was rotated around each axis of anatomical frame with its ends pointing to anatomical landmarks. This method did not rely on posture assumptions and the IMUs were calibrated to be aligned with the real anatomical axis of the segment. However, ALI required not only special devices but also longer setup time and more professional staff to perform the calibration. ALI is performed one by one for all the body segments involved. For each body segment, the staff needs to point both ends of the caliper on anatomical landmarks and rotate the caliper around different axes of the segment.

JCI utilized the limited Degree of Freedom (DoF) for joints such as the knee and elbow, to realize the anatomical calibration from arbitrary motions [15]. A complete review of researches applying joint constraints could be found in [9]. However, the usage of JCI was limited to the low DoFs joints (DoF < 3). Furthermore, two sensors on the links connected by the joint were needed so it was not suitable to calibrate only one sensor.

MBM modeled the body as multiple rigid segments which were linked together with rotational joints in a chain [16]. IMUs were firmly attached to each segment with known distances to both ends of the segment. An Extended Kalman Filter (EKF) or optimization then estimated the orientation between the IMUs and the body segments [17]. MBM could realize anatomical calibration from predefined motions [2] or arbitrary motions [17]. However, the orientation estimation through EKF and the involvement of a magnetometer may lead to errors in calibration. Besides, the requirement for all segment lengths and IMUs’ relative positions on the segments increased the measurement items during the experiment, thus could result in longer setup time. Moreover, the MBM was not applicable when IMUs were attached to only part of the segments in a kinematic chain.

Finally, FUA required the experiment participants to actively or passively perform predefined posture or movement such as flexion/extension, abduction/adduction and circumduction, and determined the rotation matrix between the coordinates frames of the sensor and the body segment through the gravity component of acceleration and the angular velocity along the anatomical axis. Besides, FUA could also be performed with the static sitting, inclined and lying postures [18]. FUA was extensively used in the research requiring the estimation of joint angle for the lower limbs [13,19,20] as well as the upper limbs [10,21,22]. Although FUA was the most popular technique for anatomical calibration, its accuracy was significantly limited to the accuracy of the predefined motion performed by the subject.

All the anatomical calibration methods except JCI required performing extra predefined movements or measurements during the experiment. Therefore, once an experiment was done without anatomical calibration, the researchers could not retrieve the alignment through post-processing on the experimental data. Although JCI did not need special measurement during the experiment, its usage was limited to joints with DoFs less than 3.

So, how can we retrieve anatomical calibration through post-processing even if this calibration was not explicitly included in the experiment protocol? Inspired by FUA, it would be possible if utilizing the motion constraints, such as the limited range of movement outside sagittal plane in walking test and sit-to-stance (StS) test. Fortunately, these tests were popularly included in various test batteries, for example Tinetti test [23], Senior fitness test (SFT) [24], and Short Physical Performance Battery (SPPB) [25]. The problem, however, was that the alignment retrieved from normal motion tests was the alignment between the IMU sensors and the whole body, instead of the body segments. This difference would affect mainly the lower limbs, for example if the person walked toe-in or toe-out. However, aligning the sensor coordinates frames to the whole body instead of specific body segments could be beneficial when motion projection on anterior-posterior (AP) and medial-lateral (ML) directions were needed.

**Figure 1:**
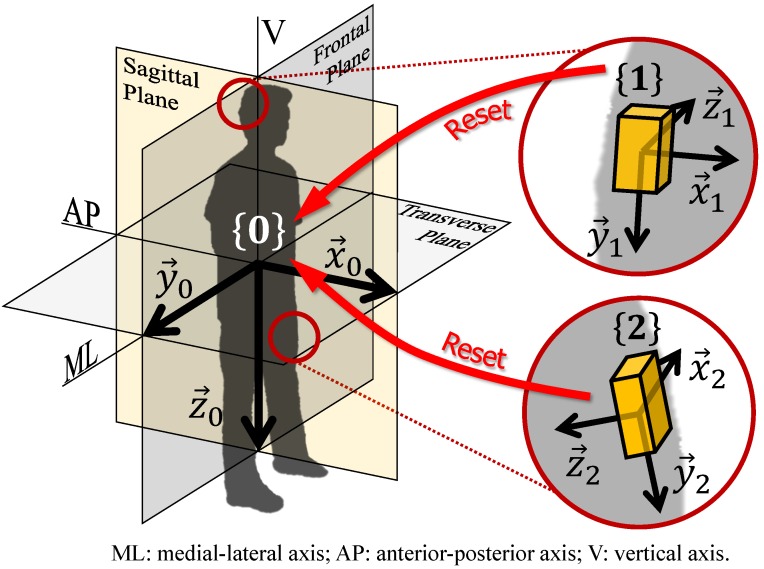
Anatomical calibration.

In this paper, the authors propose an anatomical calibration method to align the axis of the sensor coordinates frame to the axis of the “whole body” coordinates frame (Figure 1), through post-processing the data taken from quiet stance and standard motion tests whose motion is mainly on sagittal plane and has large range of movement such as walking and StS.

Furthermore, experiments are conducted to examine the feasibility of proposed method. We want to verify that proposed algorithm is robust to different sensor positions and motion types, and that after several repetitions the reset coordinates frame will approach to a stable frame which aligns with the plane of the motion. More specifically, the objectives of the experiment are:

Objective 1 Check the alignment between coordinates frames of multiple sensors attached to frontal/back and lateral side on the same body segment;
Objective 2 Check the alignment between coordinates frames of different sensors on different body segments when these segments move together as a rigid body;
Objective 3 Verify if the reset coordinates frame will approach to a stable frame after several repetitions (Gradualness);
Objective 4 Verify if the coordinates frames extracted from different repetitions are similar (Test-retest reliability);
Objective 5 Check the alignment between coordinates frames estimated from different types of anatomical calibration test, where special emphasis was put on the comparison between the alignment matrix estimated from standard motion tests and from the movement in traditional anatomical calibration.

## 2. Materials and Methods

### 2.1. Anatomical Calibration Algorithm

To measure acceleration, angles, and angular velocities by using inertial measurement systems, it is important to refer to the same reference frame. In this case, since our objective is to analyze human motion, we have to find the rotation matrix ${}_{i}^{0}R$ from {i} to {0}, where {i},i∈{1,2,3,...} is defined as the coordinates frame of IMU sensor and {0} represented the coordinates frame of the whole body, see Figure 1. The notation in this work is taken from [26]. {0} is defined as ${\vec{x}}_{0}$ axis (anterior-posterior axis) pointing forward, ${\vec{y}}_{0}$ axis (medial-lateral axis) pointing to the right, and ${\vec{z}}_{0}$ axis (vertical axis) pointing down. To calculate ${}_{i}^{0}R$ we need to find ${}^{i}{\vec{x}}_{0}$, ${}^{i}{\vec{y}}_{0}$, ${}^{i}{\vec{z}}_{0}$, the representation of each axis of {0} with respect to {i}.

(1) $${}_{i}^{0}R={{}_{0}^{i}R}^{T}={\left[\begin{array}{ccc} {}^{i}{\vec{x}}_{0} & {}^{i}{\vec{y}}_{0} & {}^{i}{\vec{z}}_{0} \end{array}\right]}^{T}$$

The unit vector ${}^{i}{\vec{z}}_{0}$ can be easily found through the acceleration asking the subject to stand still with both legs (quiet stance). According to the definition of {0}, ${}^{i}{\vec{z}}_{0}$ is along the direction of gravity, while during quiet stance the gravity is the main component of acceleration with respect to the {i} (${}^{i}\vec{a}$). Therefore ${}^{i}{\vec{z}}_{0}$ can be estimated from Equation (2). We apply a Median filtering of the data so that ${}^{i}{\vec{z}}_{0}$ will not be affected by eventual movements of the subject during this phase of calibration.

(2) $${}^{i}{\vec{z}}_{0}=\frac{median({}^{i}\vec{a})}{\left|\left|median({}^{i}\vec{a})\right|\right|}$$

However, it is difficult to define ${}^{i}{\vec{x}}_{0}$ and ${}^{i}{\vec{y}}_{0}$, i.e., the anterior-posterior (AP) and medial-lateral (ML) directions on the transverse plane, see Figure 1. Similar to ${}^{i}{\vec{z}}_{0}$, we need a proper reference vector to decide the direction of ${}^{i}{\vec{x}}_{0}$ or ${}^{i}{\vec{y}}_{0}$.

One possibility is to utilize the geomagnetic field, which is constant in direction within the experiment space, to act as a reference vector to correct the horizontal heading of the sensor [21]. However, most experiments are conducted indoor where the geomagnetic field is weakened and distorted by various sources of interference, for example the cage of reinforcement of the building, the metal parts on furniture and flooring, or the power supply lines.

In our algorithm we propose to use acceleration to find ${}^{i}{\vec{x}}_{0}$ and ${}^{i}{\vec{y}}_{0}$. Therefore, the presence of distorted geomagnetic field will not influence the results. Furthermore, the presence of gravity acceleration components on the other axis will allow us to determine the normal unit vector of the sagittal plane even if the motion during the calibration is slow.

Our anatomical calibration algorithm uses the acceleration during the movements with the following characteristics:

1. At least 80% on the sagittal plane;
2. At least 80% rotation around the medial-lateral axis (pitch rotation);
3. Range of motion over 30 degrees.

The acceleration of the movements which meet the requirements above should be distributed around the sagittal plane in {0}. Besides, the rotation between the sensor’s coordinates frame {i} and whole body frame {0} should be around the ${\vec{y}}_{0}$ axis. Therefore, after rotating the coordinates system from {0} to {i}, the 3 dimensional distribution of the acceleration should be about on a 2 dimensional plane across the origin point.

Our concept is that the predominant plane of acceleration could estimate the sagittal plane with respect to {i}, therefore the normal vector ${}^{i}\vec{n}$ of the predominant plane can be regarded as the representation of the medial-lateral axis.

(3) $${}^{i}{\vec{y}}_{0}\approx \pm^{i}\vec{n}$$

**Figure 2:**
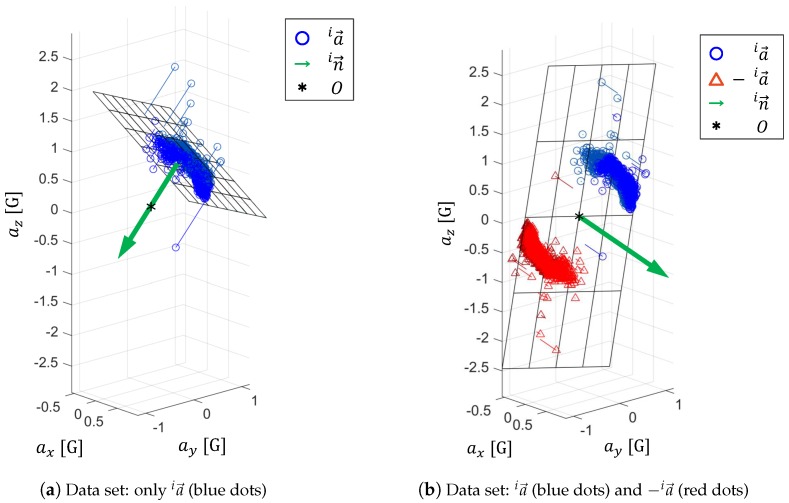
Estimated sagittal plane and normal vector extraction from PCA, adding origin symmetric data ${-}^{i}\vec{a}$ into the data set of PCA calculation helps to improve the sagittal plane estimation.

The calculation of the predominant plane and the normal vector ${}^{i}\vec{n}$ is done by Principal Component Analysis (PCA), where the first two principal components represent the predominant plane while the 3rd component is ${}^{i}\vec{n}$. The data set for PCA calculation includes both the acceleration data (${}^{i}\vec{a}$) and their origin symmetric data (${-}^{i}\vec{a}$), see Figure 2b. The reason for adding ${-}^{i}\vec{a}$ is to restrict the predominant plane to crossing the origin. Otherwise error in fitting may occur, like in Figure 2a.

As shown in Equation (3), ${}^{i}{\vec{y}}_{0}$ could be either ${}^{i}\vec{n}$ or ${-}^{i}\vec{n}$, but by the definition of Y axis in {0}, ${}^{i}{\vec{y}}_{0}$ should be along the medial-lateral axis pointing to the right side of the body. Here for simplicity, we use the relationship between the extracted normal vector and the sensor axis to determine whether ${}^{i}{\vec{y}}_{0}$ equals to ${}^{i}\vec{n}$ or ${-}^{i}\vec{n}$.

It is possible that the normal unit vector ${}^{i}\vec{n}$ of the estimated sagittal plane is not perpendicular to the vertical axis ${}^{i}{\vec{z}}_{0}$ obtained previously from the gravity. Therefore, a cross multiplication is done between ${}^{i}\vec{n}$ and ${}^{i}{\vec{z}}_{0}$. The product is perpendicular to both vectors and we assume that it represents the anterior-posterior axis ${}^{i}{\vec{x}}_{0}$. Finally, a cross product between ${}^{i}{\vec{z}}_{0}$ and ${}^{i}{\vec{x}}_{0}$ allow us to obtain the ${}^{i}{\vec{y}}_{0}$ direction.

(4) $${}^{i}{\vec{x}}_{0}={}^{i}\vec{n}\times {}^{i}{\vec{z}}_{0}$$

(5) $${}^{i}{\vec{y}}_{0}={}^{i}{\vec{z}}_{0}\times {}^{i}{\vec{x}}_{0}$$

The ${}^{i}{\vec{x}}_{0}$, ${}^{i}{\vec{y}}_{0}$, ${}^{i}{\vec{z}}_{0}$ are combined into the rotation matrix ${}_{i}^{0}R$ using Equation (1) and the matrix can be used to reset the sensor data $\vec{{}_{i}d}$ of gyroscope, accelerometer, or magnetometer from {i} to {0}.

(6) $${}^{0}\vec{d}=\left({}_{i}^{0}R\right)\left({}^{i}\vec{d}\right)$$

### 2.2. Experiment Setup

**Figure 3:**
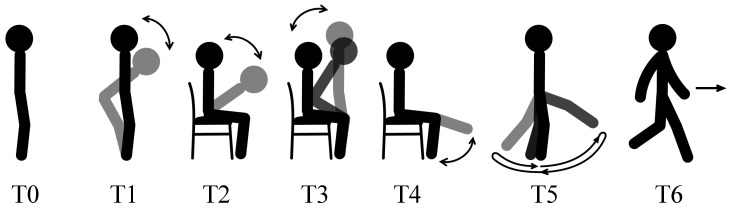
Tests in the experiment of anatomical calibration.

The protocol of the experiment included the following 7 tests (see also Figure 3):

T0 Quiet standing for 20 s, only once;
T1 Bowing 5 times while standing. We asked the subjects to keep their head, chest and waist as a rigid body;
T2 Bowing 5 times while sitting. We ask the subjects to keep the head, chest and waist as a rigid body;
T3 Standing up and sitting down 5 times on a chair. We ask the subjects to keep the arms across the chest;
T4 Lifting shanks while sitting, first left shank for 5 times then right shank for 5 times. We ask the subjects to keep the shank and foot as a rigid body;
T5 Swinging legs while standing, first left leg for 5 times then right leg for 5 times. We ask the subjects to keep the thigh, shank and foot as a rigid body;
T6 Walking straight for 7 m at the preferred speed.

Except T0, all the other tests are performed 5 times.

T0 is static test designed for extracting the vertical axis ${}^{i}{\vec{z}}_{0}$, while T1 to T6 are motion tests for estimating the sagittal plane and extracting the medial-lateral axis. The requirement in T2, T4 and T5 to keep the moving body segments as a rigid body is for Objective 2 and is not fundamental for anatomical calibration. Among the motion tests, T3 and T6 are standard motion tests included in various test batteries, while the rest are used in normal anatomical calibration procedure. It is important to point out that the motion tests for the proposed algorithm could include any test with movements on the sagittal plane, mainly with pitch rotations, and with significant range of motion.

Five young male subjects were recruited from the students of our laboratory and participated in the experiment. The subjects were chosen for having variety on height, weight and BMI, see Table 1. We performed the experiment with different subjects just to be sure that the calibration results are not biased by the particular position of the sensors on a single subject and motion during the test. We want to highlight here that we focus on the evaluation of the calibration algorithm which resets the coordinates frames of the sensors and not to the particular motion analysis of the subjects.

### 2.3. WB-4R Inertial Measurement Unit

**Figure 4:**
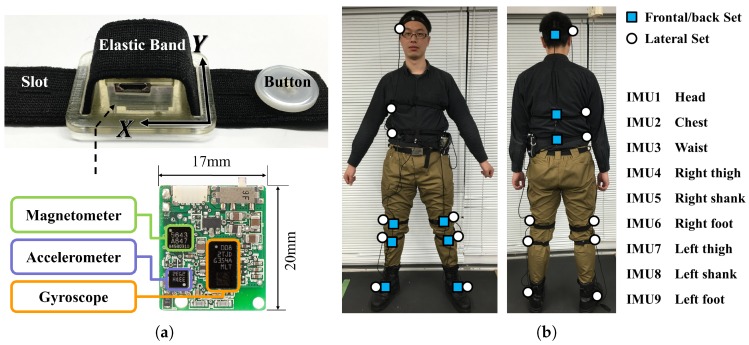
Sensor system. (**a**) WB-4R Inertial Measurement Unit (IMU); (**b**) Sensor placement. The yellow circles represent the Frontal/back Set, which was attached to the frontal side of lower limbs and the back side of trunk and head. The white circles represent the Lateral Set, which was attached to the lateral side of the body.

The IMU sensor used in the experiment is named WB-4R (Waseda bioinstrumentation 4R), which is a compact and light-weighted (17 mm × 20 mm × 8 mm, 3.9 g) Inertial Measurement Unit (IMU) containing a 3-axis accelerometer, a 3-axis gyroscope and a 3-axis magnetometer. The detailed characteristics of WB-4R sensor can be found in the reference [27]. As shown in Figure 4a, the WB-4R is attached to an elastic band which helps to tighten the sensor on human body. The elastic band has a button and slots on it, which allows fast and easy setup for experiment.

In this experiment, we used two sets of WB-4R (in total 18 IMUs) on the body of the experiment participant. Each set of sensors contained nine WB-4R IMUs placed on the head, chest, waist, both thighs, both shanks and both feet (Figure 4b). The ranges of accelerometers were set differently for the IMUs on upper body (±4 G, 1 G equals to the magnitude of local gravity acceleration) and lower body (±8 G), while the ranges of all gyroscopes were set to ±400 deg/s. One set called Frontal/back Set was attached to the front and back side of the body, while another set called Lateral Set was attached roughly to the lateral side of the body. The two sensors from different sets but on the same body segment were striped with single elastic band to make them at the same height when attached to human body. Each set of sensors were connected to a central board which synchronized the sensors through CAN bus and transmitted the data to a personal computer via Bluetooth (class 1, v2.1). The data from the two sets were aligned by removing the offset estimated from the cross correlation of the acceleration after anatomical calibration. All the sensors had been calibrated before the experiment to correct the offset, gain and misalignment matrix.

For different test from T1 to T6, the body segments where the motion is mainly on the sagittal plane and with enough range of movement are different. Table 2 shows the tests that can be used to reset a specific sensor.

### 2.4. Data Processing

The processing of experimental data can be divided into common processing for all the objectives, and objective-specific analysis that aims to fulfill each objective.

#### 2.4.1. Common Processing

The first step of common processing is calculating ${}^{i}{\vec{z}}_{0}$ of each IMU from T0 by using Equation (2).

In the second step, all five repetitions (R1, R2, R3, R4, R5) or subsets of repetitions of each motion test (T1 to T6) are joint together to form a larger data set. Different numbers of repetitions (from 1 to 5), as well as different combinations of repetitions (for example {R1,R2,R3} and {R1,R3,R4}) are used for data set construction, since we want to check the gradualness (Objective 3) and test-retest reliability (Objective 4).

To distinguish the matrices extracted with different number of repetitions and different combinations of repetitions, we define:

**Definition** **1.** ${}_{i}^{0}R_{Tj}$: the rotation matrix, from coordinates frame {0} to {i}, extracted from all the five repetitions in Tj, where Tj represents the motion tests, i∈{1,2,...,9},j∈{1,2,...,6}.

**Definition** **2.** ${}_{i}^{0}R_{Tj,A_{k}^{m}}$: the rotation matrix, from coordinates frame {0} to {i}, extracted from a specific combination of k repetitions in Tj, k∈{1,2,...,5}. There are $C_{5}^{k}$ different combinations when extracting k out of 5 repetitions. Therefore, to distinguish them, we use the symbol $A_{k}^{m}$, where $m\in \{1,...,C_{5}^{k}\}$, to represent a specific combinations of k repetitions.

**Figure 5:**
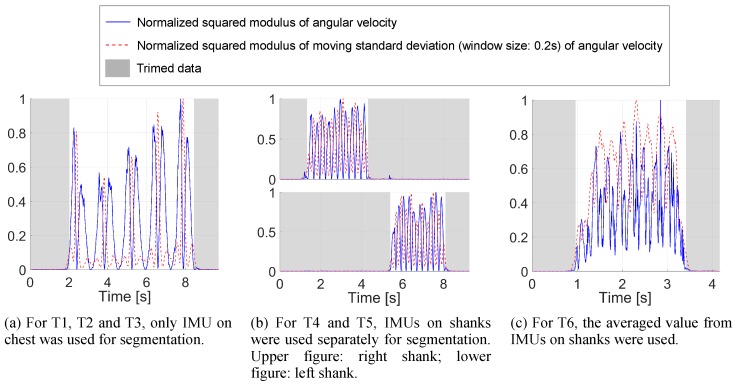
Data segmentation. Threshold at 0.1 followed by morphologically close on the binary (length: 1 s) are then applied.

Before joining into a larger data set, data in each repetition is trimmed automatically through thresholding on the normalized squared modulus of angular velocity and moving standard deviation to exclude the recording without movement, see Figure 5.

Furthermore, ${}^{i}{\vec{x}}_{0}$, ${}^{i}{\vec{y}}_{0}$, and the rotation matrices ${}_{i}^{0}R$ are calculated from each data set constructed in the last step, by using Equations (1), (4) and (5). The calculation is limited on subsets of IMUs depending on the test, see Table 2.

Finally, each rotation matrix ${}_{i}^{0}R$ is applied to the IMU data (acceleration and angular velocity) of all motion tests, including the test where the ${}_{i}^{0}R$ is extracted from and the other tests not used for matrix generation. We define:

**Definition** **3.** $T_{ex}$: the test from which the anatomical calibration matrix is extracted, Tj in ${}_{i}^{0}R_{Tj,A_{k}^{m}}$.

**Definition** **4.** $T_{ap}$: the test on which the matrix is applied.

#### 2.4.2. Objective-Specific Analysis

For Objective 1: *Check the alignment between coordinates frames of multiple sensors attached to frontal/back and lateral side on the same body segment*, acceleration data from two IMUs on each body segment, one from the Frontal/back Set and another from the Lateral Set, are compared to calculate the correlation.

(7) $${}^{0}a_{i,Tj,s}=\left({}_{i}^{0}R_{Tj,s}\right)\left({}^{i}a_{i,Tj,s}\right)$$

(8) $$c_{i,Tj,\{s,s^{\prime }\}}=corr\left({}^{0}a_{i,Tj,s},{}^{0}a_{i,Tj,s^{\prime }}\right),s^{\prime }\neq s$$

where ${}^{0}a_{i,Tj,s}$ represents the acceleration measured with IMU *i* in sensor set *s* during test Tj, with respect to coordinates frame {i}, and ${}^{i}a_{i,Tj,s}$ is the acceleration with respect to coordinates frame {0} calculated by applying the rotation matrix ${}_{i}^{0}R_{Tj,s}$. $c_{i,Tj,\{s,s^{\prime }\}}$ is the correlation between the acceleration measured from different sides of the same body segment.

For Objective 2: *Check the alignment between coordinates frames of different sensors on different body segments when these segments move together as a rigid body*, comparison is done between the acceleration data from the IMUs on different body segments which moved together, for example right shank and right foot in T4 (sitting and lifting shank). Besides T4, data from T1, T2 and T5 are also used for the analysis because these tests are conducted with the requirement to move as a rigid body.

(9) $${}^{0}a_{i,Tj}=\left({}_{i}^{0}R_{Tj}\right)\left({}^{i}a_{i,Tj}\right)$$

(10) $$c_{\{i,i^{\prime }\},Tj}=corr\left({}^{0}a_{i,Tj},{}^{0}a_{i^{\prime },Tj}\right),i^{\prime }\neq i$$

where $c_{\{i,i^{\prime }\},Tj}$ represents the correlation between the acceleration measured from different body segments in the same test Tj.

For Objective 3: *Verify if the reset coordinates frame will approach to a stable frame after several repetitions (Gradualness)*, we checked how the result rotation matrix ${}_{i}^{0}R$ will be changed by adding one more repetition. Two types of comparison are made. One is named “incremental”, comparing the rotation matrices ${}_{i}^{0}R$ generated before and after adding one more repetition (e.g., from {R1,R3} to {R1,R2,R3}). While the other is named “to last”, comparing ${}_{i}^{0}R$ generated from a subset or a full set of repetitions (e.g., {R1,R3} and {R1,R2,R3,R4,R5}). The difference between two rotation matrices is obtained and quantified by using the angle in axis-angle representation of the 3-dimensional rotation between the two matrices. Here we focus on angle because the ${}^{i}{\vec{z}}_{0}$ of each IMU is calculated only from T0 thus will not change according to the motion tests (T1 to T6). And ${}^{i}{\vec{z}}_{0}$ is used as standard to make ${}^{i}{\vec{y}}_{0}$ perpendicular with it. Therefore, the 3-dimensional rotation between the two matrices is only around ${}^{i}{\vec{z}}_{0}$ axis.

We compare the change to the rotation matrix ${}_{i}^{0}R$ before and after adding one more repetition (named as “incremental”).

(11) $$R_{e}=\left({}_{i}^{0}R_{Tj,A_{k+1}^{m^{\prime }}}\right){\left({}_{i}^{0}R_{Tj,A_{k}^{m}}\right)}^{-1}$$

The definition of ${}_{i}^{0}R_{Tj,A_{k}^{m}}$ can be found in Definition 2, and $A_{k+1}^{m^{\prime }}$ in ${}_{i}^{0}R_{Tj,A_{k+1}^{m^{\prime }}}$ is defined by adding one more repetition to $A_{k}^{m}$ in Equation (12).

(12) $$A_{k+1}^{m^{\prime }}=A_{k}^{m}\cup \left\{a\right\}$$

where $A_{k}^{m},A_{k+1}^{m^{\prime }}\subset \left\{R1,R2,R3,R4,R5\right\},m^{\prime }\in \left\{1,...,C_{5}^{k+1}\right\},a\in \left\{R1,R2,R3,R4,R5\right\},a\notin A_{k}^{m}$. Moreover, we compare the ${}_{i}^{0}R$ generated from subsets of a single repetition and from the full set of repetitions (named as “to last”):

(13) $$R_{e}=\left({}_{i}^{0}R_{Tj,A_{5}^{1}}\right){\left({}_{i}^{0}R_{Tj,A_{1}^{m}}\right)}^{-1},m\in \left\{1,2,...,5\right\}$$

The comparison between two rotation matrices is quantified by the angle in axis-angle representation (*θ* in Equation (14)) of the 3-dimensional rotation *R* (in Equation (11)) between the two ${}_{i}^{0}R$ matrices. The larger change means more repetitions will be needed for obtaining a more accurate and reliable result.

(14) $$\left(axis,angle\right)=\left(\left[\begin{array}{c} e_{x}\\ e_{y}\\ e_{z} \end{array}\right],\theta \right)$$

For Objective 4: *Verify if the coordinates frames extracted from different repetitions are similar (Test-retest reliability)*, the ${}_{i}^{0}R$ calculated from subsets of repetitions (any 3 out of 5 repetitions for example {R1,R2,R3} and {R1,R3,R4}) are compared. Similar to the processing for Objective 3, the comparison is done with angle in axis-angle representation, see Equation (14).

(15) $$R_{e}={(}_{i}^{0}R_{Tj,A_{3}^{m}}){{(}_{i}^{0}R_{Tj,A_{3}^{m^{\prime }}})}^{-1},m\neq m^{\prime }$$

For Objective 5: *Check the alignment between coordinates frames estimated from different types of anatomical calibration test*, rotation matrices ${}_{i}^{0}R$ generated by different types of tests for the same IMU sensor are compared with each other. ${}_{i}^{0}R$ of each IMU can be generated independently by three tests, see the columns in Table 2. The comparison is done also with angle in axis-angle representation.

(16) $$R_{e}={(}_{i}^{0}R_{Tj}){{(}_{i}^{0}R_{Tj^{\prime }})}^{-1},j\neq j^{\prime }$$

Except Objective 3 and Objective 4, all five repetitions of each test are used for rotation matrix generation.

## 3. Results

### 3.1. Objective 1

For Objective 1: *Check the alignment between coordinates frames of multiple sensors attached to frontal/back and lateral side on the same body segment*, acceleration data from two IMUs on each body segment, one from the Frontal/back Set and another from the Lateral Set, are compared and the correlation between the two IMUs is calculated.

**Figure 6:**
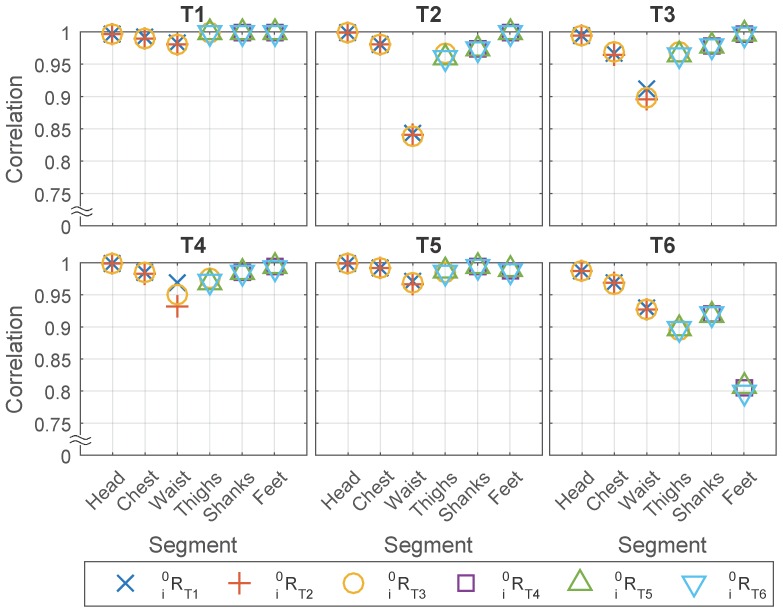
Acceleration correlation between sensors on different sides of the same segment, with different $T_{ex}$.

The following three factors are thought to be related to the correlation and averaged RMS difference: $T_{ex}$, $T_{ap}$, and the body segments where the sensors are attached to. Figure 6 shows the relationship between these three factors with the correlation.

In general, the results show that under most circumstances, the correlation is high (over 0.95) between the two sensors placed on the frontal and lateral sides of the same body segment. In some of the cases, however, the correlation is relative lower (less than or equal to 0.95) and the minimum is at around 0.8, see Figure 6.

More specifically, we find that the correlation and averaged RMS difference are not related with $T_{ex}$ but highly related with $T_{ap}$ and body segment, which suggested that first the results will be similar regardless of which type of motion is used for anatomical calibration, and second the motion of some body segments in some of the tests are not the same on the frontal and lateral side.

For the first point, we found that even if the matrices are generated from the test where two IMUs had lower correlation for example feet in T6, the result in Figure 6 is similar with the matrices generated from other tests such as T4 and T5. Since the $T_{ex}$ is not related to the correlation, it is averaged to compress Figure 6 to Table 3.

For the second point, ideally we suppose the body segments to be rigid bodies so that the motion on different sides will be the same. However, in reality the body segments are not ideal rigid bodies therefore the motion on the lateral and frontal sides suffered to different extents from the soft tissue artifact such as the fat under the sensor on lateral waist or the muscle under the sensor on frontal thighs, and/or the bending of body segment such as foot in walking test (T6). The head, which is the most rigid body segment in our experiment, achieves that highest correlation (all over 0.995, except 0.986 in T6). While the correlation is relatively lower for segments prone to soft tissue artifacts for example the waist, or during the tests having fast movement for example sit-to-stand (T3) and walking (T6). The waist in T2, T3, T4 is affected by the artifact of soft tissue underneath the lateral sensor when sitting on/onto a chair, and the lower limbs in T6 are affected by soft tissue artifacts because of muscle contraction under the sensors.

**Figure 7:**
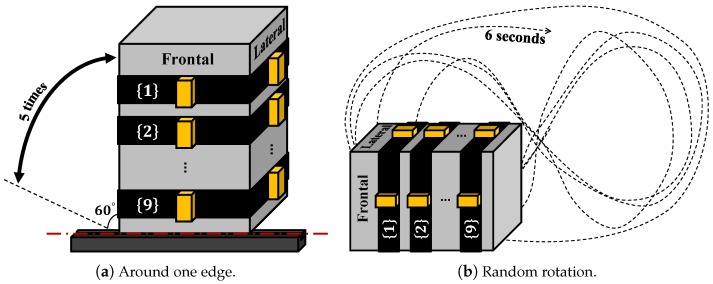
Experiment setup with an aluminum box.

To examine how much correlation can be with a rigid body, the same sets of sensors were placed onto the frontal and lateral sides of an aluminum box and two types of tests (rotate around one edge, Figure 7a; rotate randomly, Figure 7b) were conducted with 5 repetitions. The five repetitions of rotation around one axis were then used for generating the anatomical calibration matrix and applied on rotation around one axis test and random rotation test, repeating the same data processing used for human subjects. The results (Table 4) show high correlation between the frontal lateral sensor pairs, especially when rotation around one axis (correlation larger or equal to 0.998). The lower acceleration correlation (around 0.97) in the random rotation is due to centrifugal acceleration, because the distances from the two IMUs to the rotation axis are neither constant nor equal. This can be seen from the high correlation on angular velocity in Table 5.

### 3.2. Objective 2

For Objective 2: *Check the alignment between coordinates frames of different sensors on different body segments when these segments moves together as a rigid body*, the purpose through this objective is to confirm that after the anatomical calibration, the coordinates frames of different body segments are closely aligned. In order to verify that, we asked the experiment participants to move their upper body in T1 and T2 and their lower limbs in T4 and T5 as much as they could as one rigid body, to obtain the similar movement among the body segments. Suppose the coordinates frames of different segments are aligned, the projection of the movement to these frames should be highly correlated.

However, the accuracy and reliability of this verification are limited to two factors: first, the relative movement between segments because the participants fail to keep multiple segments as one rigid body; second, the difference of centrifugal acceleration because the distances from different sensors to the rotation axis are not equal.

To minimize the impact of these two factors on the verification of our objective, we exclude waist and feet from the analysis and focused on the correlation between the head and chest, and between the thighs and shanks. Also, T4 is removed from the analysis of Objective 2 because it involves only the comparison between shanks and feet. The first reason to exclude waist and feet is because we find that participants are particularly difficult to move the waist and feet with the rest part of upper body and lower limbs, respectively, together as one rigid body. The waist is easily deformed in T1 and T2 when bowing forward, while the feet tends to rotate with respect to the shank in order to avoid the feet from hitting the ground when swinging shanks or legs in T4 and T5. The second reason to exclude waist and feet is to minimize the difference of centrifugal acceleration. The waist is much closer to the rotation axis when bowing forward compared with the head and chest, and the feet are much farther to rotation axes when swinging the shanks (T4) or swinging the legs (T5) compared with the sensors placed on thighs and shanks, between which the distance are only about 15 cm as shown in Figure 4b.

**Figure 8:**
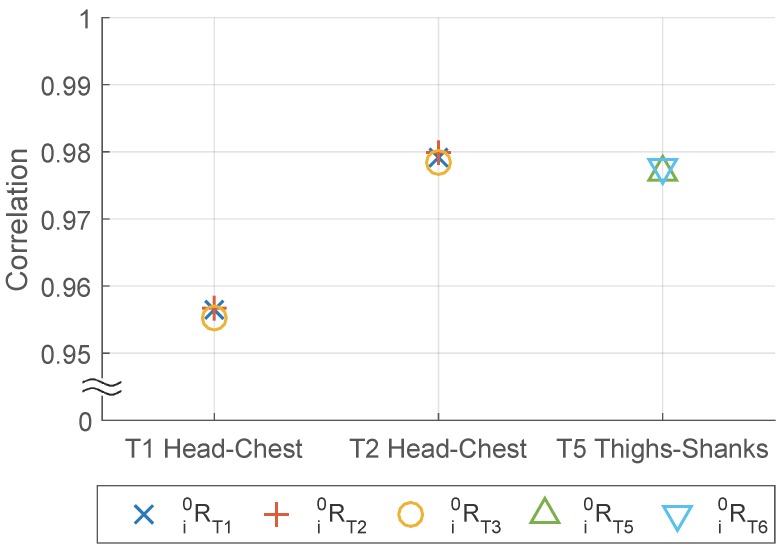
Acceleration correlation between different body segments, with different $T_{ex}$.

Similar to Objective 1, we calculate the correlation and check the impact of $T_{ex}$, $T_{ap}$ and body segments pairs on it, while the difference is in Objective 2 we check the pairs of sensors placed on different body segments from the same IMU set instead of on the same body segment from different sensor sets. From the result shown in Figure 8 and Table 6, we find the similar conclusion in Objective 1: correlation is related strongly with $T_{ap}$ and body segment but not with $T_{ex}$.

Furthermore, concerning the frontal or lateral placement of the sensor, a paired *t*-test is performed in Table 7. The results suggest that the two sets have similar performance on correlation. Although for head-chest body segment pair the Lateral Set is significantly better than the Frontal/back Set in the paired *t*-test, the difference in averaged correlation value is marginal.

### 3.3. Objective 3

For Objective 3: *Verify if the reset coordinates frame will approach to a stable frame after several repetitions (Gradualness)*, the result can suggest how many times of repetitions are needed for different body segments and different $T_{ex}$ to obtain a higher precision.

**Figure 9:**
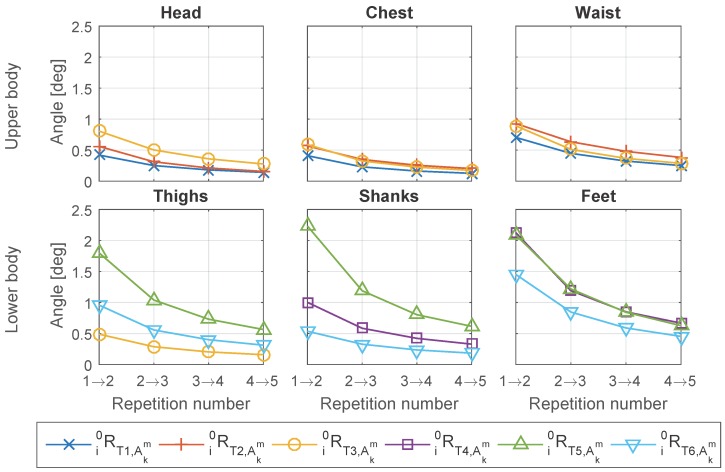
Averaged error angle when adding one more repetition (“increment”).

The “incremental” comparison shown in Figure 9 suggests that the rotation matrix ${}_{i}^{0}R$ is gradually converging to zero, and the change in ${}_{i}^{0}R$ decreases as increase of the number of repetitions. Besides, the change caused by adding repetitions is small. The maximum of averaged error angle is less than 2.5° for all the $T_{ex}$ and body segments, and after including three repetitions the change caused by adding one more repetition in ${}_{i}^{0}R$ is less than one degree.

**Figure 10:**
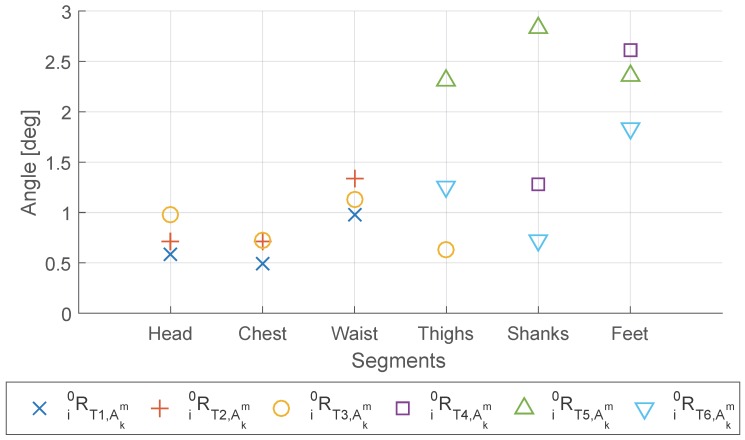
Averaged error angle between using only one or all the five repetitions (“to last”).

Through the “To last” comparison shown in Figure 10, we find that the lower limbs (thighs, shanks and feet) showed relatively larger change compared with the upper body, although the maximum change for all segments is still less than 3 degrees. The larger change can be caused by larger variance between different repetitions due to the lower motion repeatability, or by the larger error in matrix estimation from each repetition due to the more divergent distribution of acceleration data. The waist and feet have the highest change within the upper body and the lower limbs, respectively.

Besides, from Figure 10 we notice that the variance between different $T_{ex}$ is relatively large compared with their mean, especially for the lower limbs. Interestingly, we find that for all the segments in lower limbs, T6 (7 m walking) has much smaller change compared with T5 (standing and swing legs), when increasing the number of repetitions from 1 to 5. Although compared with T5 the acceleration data distribution of T6 is more divergent and apart from being on one plane for a single repetition, T6 has much less variance between different repetitions and therefore has less change when adding one more repetition.

Concerning the frontal or lateral IMU placement on the same body segment, paired *t*-test is conducted between the Frontal/back Set and Lateral Set for the ${}_{i}^{0}R$ generated from different number of repetitions and different combinations of repetitions. The result of paired *t*-test is shown in Table 8. The results showed that the Frontal/back Set is better for the upper body, having higher correlation, while the Lateral Set is better for the lower limbs. The reason could be for the upper body the sensors placed on the lateral side of the body are more vulnerable to the soft tissue artifact as the Frontal/back Set is placed close to the bone, and for the lower body the sensors on the lateral side of the body suffered less by the muscle activation because for the motion mainly in the sagittal plane the frontal muscle groups are activated and affected the measurement of the sensor placed on the frontal side of the leg. The exceptions, however, are the thighs calibrated in T3 (chair standing), for which the Frontal/back Set is significantly better (*p* value less than 0.001) than the Lateral Set. The reason is that the lateral side of the thighs deform when sitting onto the chair, therefore in this specific case the sensor on the frontal side of the thigh performs better than on the lateral side.

### 3.4. Objective 4

For Objective 4: *Verify if the coordinates frames extracted from different repetitions are similar (Test-retest reliability)*, ${}_{i}^{0}R$ calculated from different subsets of three repetitions are compared. The comparison is quantified with angle in axis-angle representation.

The results are summarized in Table 9, by averaging among the different subsets and IMU sets. From the result, we find that the T5 and the feet had averaged difference larger than 1 degree, which means that they are less reliable therefore more repetitions will be needed in order to improve the reliability. This result agrees with the one we obtained in Objective 3 where T5 and the feet have relatively larger error angle in Figure 10.

Concerning the IMU placement on different side of the same body segment, the result from Objective 4 is the same as Objective 3. The Frontal/back Set is more reliable for the upper body, while the Lateral Set is more reliable for the lower body except the thighs in T3, see Table 10.

### 3.5. Objective 5

For Objective 5: *Check the alignment between coordinates frames estimated from different types of anatomical calibration test, where special emphasis was put on the comparison between the alignment matrix estimated from standard motion tests and from the movement in traditional anatomical calibration*, rotation matrices ${}_{i}^{0}R$ generated by different types of tests for the same IMU sensor are compared with each other.

From Table 11, we find that in general the error angles between ${}_{i}^{0}R$ estimated from different $T_{ex}$ are higher than the ones we obtained in Objective 3 and Objective 4. The reason is because the predominant motion plane differ slightly for different tests. For example, the change of distance and angle between the two feet in sit-to-stance test (T3) would affect the predominant motion planes of both thighs. Therefore, the estimation of sagittal planes would be affected and lead to error compared with T4 and T5.

For the upper body, especially for the head and chest, the difference between the ${}_{i}^{0}R$ generated from different tests are small. The waist shows slightly larger difference between T1 and T2 or T3, which is because T2 and T3 includes sitting while T1 is standing.

For the lower limbs, we find that the thighs bore large difference (over 10 degrees) between T3 and T5 or T6, while the difference between T5 and T6 is relatively low (less than 5 degrees). The shanks have better results, having all the differences lower than 5 degrees except between T5 and T6. The feet, however, has the differences are ranging from 8 to 10 degrees for all the test pairs (T4-T5, T4-T6, T5-T6), which means the feet are sensitive to the $T_{ex}$ thus anatomical calibration for feet is less reliable.

Finally, the alignment matrices estimated from standard motion tests (T3 and T6) is compared with the ones obtained from movement specially designed for anatomical calibration (T1, T2, T4, T5). For upper body (head, chest, waist), T3 can be competitive with T1 and T2, with the averaged error angle in maximum 5.39 degree. For thighs and shanks, T6 can be competitive with T4 and T5, with the averaged error angle in maximum lower than 5.12 degree. However, it seems to be not desirable to use T3 for thighs’ anatomical calibration. Besides, for feet, the error angles are in average larger than 8.5 degree between all the pairs of T4, T5 and T6, which means the ankle joints were so mobile that the feet were difficult to be accurately calibrated, even with the designed movement for anatomical calibration.

Therefore, we recommend to use StS test (T3) for anatomical calibration of head, chest and waist, and use walking test (T6) for anatomical calibration of thighs and shanks. In such cases, the error angle would be around and less than 5 degrees in average compared with the tests designed for anatomical calibration.

In this work, the error angle is always around the vertical axis (${\vec{z}}_{0}$), because the motion tests (T1 to T6) determine only the horizontal axes (${\vec{x}}_{0}$ and ${\vec{y}}_{0}$). Therefore, its impact on data analysis depends on the relationship between the predominant motion plane and the plane on which the analysis is done. Specifically, the impact of error angle is negligible when the two planes are identical, but the impact could become considerably large when the two plane are perpendicular. For example, let us suppose that the movements are predominantly on sagittal plane, and the error angle between the sagittal plane and the estimated ${\vec{x}}_{0}$-${\vec{z}}_{0}$ plane is 15 degree. If we want to analyze the motion on sagittal plane, since the error angle is always around the vertical axis, over 97% ($cos(15^{\circ })$) of the motion amplitude on sagittal plane will still remain on the estimated ${\vec{x}}_{0}$-${\vec{z}}_{0}$ plane. So even the error angle is close to the upper boundary of error angle in Table 11, it does not undermine the analysis. However, if we want to analyze the same motion on frontal plane, we will find that at maximum about 26% ($sin(15^{\circ })$) of the predominant movements on sagittal plane is projected to the estimated ${\vec{y}}_{0}$-${\vec{z}}_{0}$ plane, leading to a significant error in the analysis on frontal plane. Since for most of the human movement, especially for lower limbs, the predominant motion plane is the sagittal plane, we recommend to use the anatomical calibration method for the analysis on sagittal plane.

## 4. Discussion

From Objective 1 and Objective 2, the correlations are overall high between sensors on different sides of the same body segment, and between the sensors on the different body segments when they are moving together, except for the waist and the feet. The correlation is not related with which test is used for generating the alignment matrix ($T_{ex}$) but related with on which body segment and in which test the matrix is applied ($T_{ap}$).

The verification of Objective 1 and Objective 2 is limited by the artifact and the bending of body segments. However, changing the perspective, this means that, for some of the body segments in some tests, the movements measured by sensors on different sides of body are different. Therefore, the lateral sensor cannot replace the frontal sensor by simply resetting its coordinates frame at the beginning, or vice versa. The segments affected are the easily bendable segments, such as waist and feet, and the tests in which the frontal and lateral movements are different are the ones including bending of these segments or strong muscle activation, such as sit-to-stand (T3) or walking (T6).

Concerning the sensor placement on different sides of the same body segment, we find (Objective 2 to Objective 5) that in general the Frontal/back Set is better for the upper body, while the Lateral Set is better for the lower body.

Moreover, we verify that not too many repetitions were needed for converging the alignment matrix to a stable stage, from three repetitions the matrix changes in average for only less than 1 degree when adding one more repetition (Objective 3), and the averaged difference between the matrices generated from different combinations of three repetitions are less than 2.1 degrees and even less than 1 degree excluding T5 and both feet (Objective 4). More specifically, we find that in more natural movements, such as sit-to-stand (T3) and walking (T6), while the distribution of the accelerations is not a single neat plane, the repeatability of these motions are high. Therefore, fewer repetitions are needed to stabilize the alignment matrix compared with less disperse but more unnatural motions, such as lifting shank in T4 and swinging leg in T5.

Finally, because the motion is performed by humans, the estimated “sagittal plane” or the predominant plane of acceleration distribution in different tests are different. From Objective 5 we know that this difference is close to or less than 5 degree, in exception of the ones related with feet or T3 thighs. Including them, in the extreme case the difference between matrices can be in average as large as 15 degrees. As discussed at the end of Section 3.5, the impact of this error depends on the motion as well as the analysis. For example, if the predominant movements are on sagittal plane, the analysis on sagittal plane will still be accurate even with the error in coordinates frame alignment, but the analysis on frontal plane may be heavily affected. In fact, in Objective 1 and Objective 2 we observe no large impact due to the error of extracted matrix.

Although in this work the experiments were conducted with healthy young subjects, the algorithm can be applied to pathologic or elderly subjects. However, as mentioned in Section 2.1, the motion for this anatomical calibration method must satisfies the three characteristics (mainly pitch rotation, on sagittal plane, and having at least 30 degrees of motion range). If the motion of pathologic or elderly subjects does not satisfy the three requirements in normal motion tests, instead of normal motion tests, the motion for anatomical calibration should be the predefined motions such as bowing or swinging the leg. Sometimes some certain types of motion might be difficult to do by the subject. With our approach, the anatomical calibration can be done with an assistant helping the subjects to move their body at a very low speed, because this algorithm depends on acceleration (including gravity) instead of angular velocity, like in previous studies [10,13,19,20]. Besides, during quiet stance, the pathologic subjects may lean towards one side of body and the elderly subjects may be humpbacked. In such cases, the “whole body” coordinates frame estimated from anatomical calibration may be largely misaligned with the coordinates frames of each body segments.

## 5. Conclusions

To conclude, in order to conduct anatomical calibration through post-processing the data taken from quiet stance and any standard motion test whose motion is mainly on sagittal plane and has large range of movement, an anatomical calibration method is proposed to align the axes of the sensor coordinates frame to the whole body coordinates frame.

Furthermore, experiments which are conducted with two sets of IMUs placed on frontal/back and lateral sides of head, trunk and lower limbs show that after the proposed anatomical calibration the coordinates frames are closely aligned for sensors placed on different sides of the same body segment.

Moreover, the repeatability and convergence of the proposed method are verified with multiple test repetitions. Finally, the anatomical calibrations performed with motion tests (StS and walking) are similar to the ones obtained with movements specifically designed for upper and lower body anatomical calibration respectively, except for feet.

Compared with the other researches in the literature, the advantage of the proposed anatomical calibration method is that no extra calibration movement or measurement is required during the experiment. This does not only shorten the preparation time before the experiment, but also make it possible to correct the coordinates of the data taken in previous experiments when anatomical calibration is skipped. Besides, the proposed method is based on the predominant motion plane instead of the joint constraint or kinematic model. Therefore, it can be used when only a single IMU is placed on one limb. Furthermore, the method proposed requires only data from accelerometer, while the traditional methods need data from both gyroscope and accelerometer. This makes it possible to conduct anatomical calibration with extremely slow movement, for example moving the limbs of hemiplegic patients by physicians.

## Figures and Tables

**Table 1:** Anthropometric information of experimental subjects.

Subject	S1	S2	S3	S4	S5	Mean	SD
Age	27	23	23	22	26	24.2	2.2
Height (cm)	174	173	166	169	187	173.8	8.0
Weight (kg)	90	80	68	56	84	75.6	13.6
BMI	29.7	26.7	24.7	19.6	24.0	25.0	3.7

**Table 2:** Body segments of which coordinates frames can be reset.

	Upper Body			Right Leg			Left Leg		
$T_{\mathrm{ex}}$	IMU1	IMU2	IMU3	IMU4	IMU5	IMU6	IMU7	IMU8	IMU9
T1	Yes	Yes	Yes	No	No	No	No	No	No
T2	Yes	Yes	Yes	No	No	No	No	No	No
T3	Yes	Yes	Yes	Yes	No	No	Yes	No	No
T4	No	No	No	No	Yes	Yes	No	Yes	Yes
T5	No	No	No	Yes	Yes	Yes	Yes	Yes	Yes
T6	No	No	No	Yes	Yes	Yes	Yes	Yes	Yes

Yes: IMU can be reset in $T_{ex}$; No: IMU cannot be reset in $T_{ex}$.

**Table 3:** Averaged correlation between sensors on frontal/back and lateral side of the body.

	Upper Body			Lower Body		
	**Head**	**Chest**	**Waist**	**Thighs**	**Shanks**	**Feet**
$T_{\mathrm{ap}}$	**IMU1**	**IMU2**	**IMU3**	**IMU4,7**	**IMU5,8**	**IMU6,9**
T1	0.997±0.002	0.990±0.008	0.981±0.017	0.998±0.001	0.999±0.000	0.999±0.001
T2	0.998±0.002	0.980±0.011	0.841±0.287	0.962±0.028	0.974±0.024	0.998±0.002
T3	0.995±0.003	0.967±0.022	0.902±0.146	0.966±0.014	0.979±0.018	0.996±0.005
T4	0.999±0.001	0.984±0.007	0.950±0.087	0.972±0.016	0.985±0.012	0.993±0.006
T5	0.998±0.001	0.992±0.003	0.969±0.020	0.986±0.013	0.994±0.003	0.988±0.010
T6	0.986±0.013	0.968±0.013	0.928±0.042	0.896±0.044	0.920±0.032	0.803±0.069

Grey background: correlations lower than 0.95.

**Table 4:** Acceleration correlation when attaching to an aluminum box.

$T_{\mathrm{ap}}$	IMU1	IMU2	IMU3	IMU4	IMU5	IMU6	IMU7	IMU8	IMU9
Around one edge	0.998	0.999	0.999	0.998	0.998	0.999	0.998	0.999	0.998
Random rotation	0.976	0.962	0.976	0.969	0.976	0.956	0.957	0.975	0.947

**Table 5:** Angular velocity correlation when attaching to an aluminum box.

$T_{\mathrm{ap}}$	IMU1	IMU2	IMU3	IMU4	IMU5	IMU6	IMU7	IMU8	IMU9
Around one edge	0.997	0.999	0.999	0.997	0.996	0.999	0.997	0.998	0.999
Random rotation	0.998	0.996	0.998	0.996	0.997	0.988	0.993	0.996	0.989

**Table 6:** Acceleration correlation between body segments.

Segments Pair	T1	T2	T5
Head-Chest	0.956±0.037	0.979±0.015	
Thighs-Shanks			0.977±0.020

**Table 7:** Paired *t*-test on acceleration correlation between IMUs placed on different sides of body segments.

Type	Head-Chest	Thighs-Shanks
Frontal/back Set	0.966±0.028	0.977±0.019
Lateral set	0.970±0.033	0.977±0.021
*p* value	**	0.682

** *p* < 0.01.

**Table 8:** Paired *t*-test on error angles in “incremental” comparison between the IMUs placed on different sides of body segments.

	Upper Body			Lower Body		
	**Head**	**Chest**	**Waist**	**Thighs**	**Shanks**	**Feet**
Frontal/back set	0.372	0.227	0.290	0.734	0.883	1.280
Lateral set	0.390	0.430	0.833	0.629	0.658	1.080
*p* value	***	***	***	***	***	***

*** *p* < 0.001; Grey background: the smaller averaged angles of the two sets.

**Table 9:** Error angle (unit: degree), when using different subsets of three repetitions from the same test.

	Upper Body			Lower Body		
$T_{\mathrm{ex}}$	**Head**	**Chest**	**Waist**	**Thighs**	**Shanks**	**Feet**
T1	0.345±0.355	0.315±0.290	0.621±0.911			
T2	0.397±0.288	0.464±0.403	0.920±1.449			
T3	0.715±0.842	0.425±0.392	0.686±0.719	0.384±0.397		
T4					0.770±0.627	1.520±1.691
T5				1.325±1.536	1.437±1.681	1.635±2.689
T6				0.737±0.708	0.431±0.446	1.124±1.099

Grey background: angles over 1°.

**Table 10:** Paired *t*-test on error angles in Objective 4 between the IMUs placed on different sides of body segments.

	Upper Body			Lower Body		
	**Head**	**Chest**	**Waist**	**Thighs**	**Shanks**	**Feet**
Frontal/back set	0.472	0.273	0.349	0.868	1.011	1.547
Lateral set	0.499	0.529	1.137	0.762	0.748	1.305
*p* value	***	***	***	***	***	***

*** *p* < 0.001; Grey background: the smaller averaged angles of the two sets.

**Table 11:** Averaged error angle between ${}_{i}^{0}R$ matrices estimated from different tests.

	Upper Body			Lower Body		
$T_{\mathrm{ex}}$	**Head**	**Chest**	**Waist**	**Thighs**	**Shanks**	**Feet**
T1-T2	1.17±0.72	2.89±2.22	4.67±6.23			
T1-T3	1.62±0.94	3.37±2.59	5.39±5.75			
T2-T3	1.87±1.48	3.77±4.61	3.05±2.08			
T3-T5				13.66±5.50		
T3-T6				10.24±5.75		
T4-T5					4.30±3.37	8.80±6.60
T4-T6					3.69±2.06	9.84±7.35
T5-T6				4.46±3.97	5.12±4.61	8.90±7.76

Grey background: angle larger than 5 degrees.

## Abbreviations

The following abbreviations are used in this manuscript:

AC	Anatomical calibration
ALI	Direct anatomical landmark identification
AP	Anterior-posterior
BMI	Body mass index
DoF	Degree of freedom
FUA	Functional approach
IMU	Inertial measurement unit
JCI	Imposition of joint constraints
MBM	Multibody Modeling
ML	Medial-lateral
RMS	Root mean square
SFT	Senior fitness test
SPPB	Short Physical Performance Battery
StS	Sit-to-stand
WB-4R	Waseda bioinstrumentation # 4 Refined
